# Clinical implications of plasma circulating tumor DNA in gynecologic cancer patients

**DOI:** 10.1002/1878-0261.12791

**Published:** 2020-09-17

**Authors:** Lindsey M. Charo, Ramez N. Eskander, Ryosuke Okamura, Sandip P. Patel, Mina Nikanjam, Richard B. Lanman, David E. Piccioni, Shumei Kato, Michael T. McHale, Razelle Kurzrock

**Affiliations:** ^1^ Division of Gynecologic Oncology Department of Obstetrics, Gynecology, and Reproductive Sciences University of California San Diego Moores Cancer Center La Jolla CA USA; ^2^ Center for Personalized Cancer Therapy and Division of Hematology and Oncology University of California San Diego Moores Cancer Center La Jolla CA USA; ^3^ Guardant Health, Inc. Redwood City CA USA

**Keywords:** circulating tumor DNA, gynecologic cancer, liquid biopsy, matched therapy, mutation allele frequency, next‐generation sequencing

## Abstract

Molecular characterization of cancers is important in dictating prognostic factors and directing therapy. Next‐generation sequencing of plasma circulating tumor DNA (ctDNA) offers less invasive, more convenient collection, and a more real‐time representation of a tumor and its molecular heterogeneity than tissue. However, little is known about the clinical implications of ctDNA assessment in gynecologic cancer. We describe the molecular landscape identified on ctDNA, ctDNA concordance with tissue‐based analysis, and factors associated with overall survival (OS) in gynecologic cancer patients with ctDNA analysis. We reviewed clinicopathologic and genomic information for 105 consecutive gynecologic cancer patients with ctDNA analysis, including 78 with tissue‐based sequencing, enrolled in the Profile‐Related Evidence Determining Individualized Cancer Therapy (NCT02478931) trial at the University of California San Diego Moores Cancer Center starting July 2014. Tumors included ovarian (47.6%), uterine (35.2%), cervical (12.4%), vulvovaginal (2.9%), and unknown gynecologic primary (1.9%). Most ovarian and uterine cancers (86%) were high grade. 34% (*N* = 17) of ovarian cancers had *BRCA* alterations, and 22% (*N* = 11) were platinum sensitive. Patients received median 2 (range 0–13) lines of therapy prior to ctDNA collection. Most (75.2%) had at least one characterized alteration on ctDNA analysis, and the majority had unique genomic profiles on ctDNA. Most common alterations were *TP53* (*N* = 59, 56.2% of patients), *PIK3CA* (*N* = 26, 24.8%), *KRAS* (*N* = 14, 13.3%), *BRAF* (*N* = 10, 9.5%), *ERBB2* (*N* = 8, 7.6%), and *MYC* (*N* = 8, 7.6%). Higher ctDNA maximum mutation allele frequency was associated with worse OS [hazard ratio (HR): 1.91, *P* = 0.03], while therapy matched to ctDNA alterations (*N* = 33 patients) was independently associated with improved OS (HR: 0.34, *P* = 0.007) compared to unmatched therapy (*N* = 28 patients) in multivariate analysis. Tissue and ctDNA genomic results showed high concordance unaffected by temporal or spatial factors. This study provides evidence for the utility of ctDNA in determining outcome and individualizing cancer therapy in patients with gynecologic cancer.

## Graphical abstract

In gynecologic cancer patients, therapy matched to ctDNA alterations (*N* = 33 patients) was independently associated with improved overall survival (hazard ratio: 0.34, *P* = 0.007) compared to unmatched therapy (*N* = 28 patients) in multivariate analysis. Tissue and ctDNA genomic results showed high concordance unaffected by temporal or spatial factors. ctDNA may be an important tool to individualize cancer therapy in patients with gynecologic cancer.

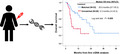

**Abbreviations**
BMI: body mass index
BRCA: breast cancer susceptibility gene
CAP: College of American Pathologists
CI: confidence interval
CLIA: Clinical Laboratory Improvement Amendments
ctDNA: circulating tumor DNA
Del: deletion
HR: hazard ratio
ID: identification number
In/del: insertion/deletion
MAF: mutation allele frequency
NR: not reached
OS: overall survival
PREDICT: Profile‐Related Evidence Determining Individualized Cancer Therapy
SE: standard error
SNV: single nucleotide variant
UCSD: University of California San Diego
VUS: variants of unknown significance

## 1 Background

In 2019, approximately 109 000 women were diagnosed with gynecologic cancer, and approximately 33 100 women died from their disease [1]. The major gynecologic cancers are ovarian, uterine, and cervical cancer, with rarer cases of vulvar and vaginal cancer. Gynecologic cancers are treated with surgery, radiation therapy, chemotherapy, or a combination of these modalities based on stage, histologic risk factors, and other tumor or patient‐specific risk factors. Despite attempts to advance therapy, recurrences and treatment failures are common, particularly for those patients diagnosed with advanced stage disease.

It is now clear that cancers are driven by specific genomic abnormalities, many of which can be targeted using existing therapies [2]. Schwaederle *et al*. [3] examined 570 phase II single‐agent studies (*N* = 32 149 patients) and found that patients who received a personalized, biomarker‐directed therapy had significantly improved outcomes and fewer deaths related to treatment toxicity. Molecular signatures have recently predicted therapeutic response in patients with gynecologic malignancies. Breast cancer susceptibility gene (*BRCA*) 1/2 mutated and homologous recombination‐deficient epithelial ovarian cancers have demonstrated dramatic responses to PARP inhibitors, *HER2*‐positive uterine serous cancers benefit from the incorporation of trastuzumab, and mismatch repair deficient or microsatellite unstable high tumors benefit from immune checkpoint inhibition with guidelines recommending MSI testing in ovarian, cervical, and vulvar cancers [4, 5, 6]. Additionally, the FDA recently approved larotrectinib as its second tumor‐agnostic approval based on tumor molecular genetics; of note, most patients in the study had cancer types where the frequency of TRK‐fusion was less than one percent, highlighting the importance of considering molecular tumor analysis broadly [7, 8].

Molecular analysis of tumor tissue is increasingly being incorporated in the development of treatment regimens for patients with solid malignancies. However, patients are often incompletely tested, or undergenotyped, for guideline‐recommended targetable mutations. For tumor tissue‐based testing, adequate samples are not always available or accessible for analysis. Even when available, tissue obtained at the time of primary surgical resection or biopsy may not reflect the current tumor molecular makeup or adequately capture tumor heterogeneity. Plasma‐derived circulating tumor DNA (ctDNA) offers a more convenient, less invasive, and real‐time option to analyze tumor for potentially actionable mutations. In an effort to better understand the value of ctDNA in the management of gynecologic cancer, we describe the molecular landscape identified on ctDNA analysis, determine concordance between ctDNA and tissue‐based analysis, and identify factors associated with survival in this cohort of gynecologic cancer patients.

## 2 Methods

### 2.1 Study patients

We reviewed the clinicopathologic and genomic information for 105 consecutive gynecologic cancer patients with ctDNA analysis who were enrolled in the Profile‐Related Evidence Determining Individualized Cancer Therapy (PREDICT, NCT02478931) trial at the University of California San Diego (UCSD) Moores Cancer Center starting July 2014. All investigations followed UCSD Internal Review Board guidelines, and consent was obtained for investigational therapies or procedures [9]. The study methodologies conformed to the standards set by the Declaration of Helsinki.

### 2.2 Circulating tumor DNA sequencing

All blood samples for ctDNA were evaluated at Guardant Health, Inc (Redwood City, CA, USA), a Clinical Laboratory Improvement Amendments (CLIA)‐certified and College of American Pathologists (CAP)‐accredited clinical laboratory. The assay sequences cancer‐associated somatic mutations in ctDNA. The panel initially included 54 genes in 2015, and it has been expanded to include 73 genes (Table S1) [10].

To assess concordance between plasma ctDNA and solid tissue biopsies, we compared frequencies of alterations in the subset of patients who had both ctDNA and tissue sequencing. All tissue DNA analysis was performed by Foundation Medicine, Inc (Cambridge, MA, USA), a CLIA‐licensed and CAP‐accredited clinical laboratory. All tissue samples were collected between July 2011 and July 2018, either at primary surgery (*N* = 31) or at recurrence (*N* = 47). The assay analyzed up to 324 genes [10, 11].

### 2.3 Outcome definitions and statistical method

Patients were described by primary disease site, histology, smoking status, body mass index (BMI), ethnicity, and number of lines of chemotherapy prior to ctDNA analysis. Categorical variables and continuous variables were compared with Fisher's exact tests and Mann–Whitney U‐tests, respectively. We included only characterized genetic alterations, excluding variants of unknown significance (VUS) and synonymous alterations. Number of ctDNA alterations and percentage of ctDNA were reported. If more than one ctDNA sample was available, we used the first sample collected. Each sample was categorized as actionable by UCSD PREDICT criteria or OncoKB criteria [12, 13]. Each patient's primary oncologist dictated which therapy a patient received. Rates of matching were reported by ctDNA and by tissue biopsy alone. Analysis was performed on patients who were prospectively or retrospectively matched. If patients were not matched, reasons for not matching were reported. Frequency and type [single nucleotide variant (SNV), amplification, or deletion, which included frameshift mutations, deletions, and insertion/deletions] of genetic alterations were then reported in the cohort of all gynecologic cancer patients and in the ovarian, uterine, and cervical/vaginal/vulvar cancer cohorts. If patients had multiple genetic alterations of the same type in the same gene, it was counted once. However, if, for example, patients had a *PIK3CA* SNV and *PIK3CA* amplification, each was counted once. All data were abstracted from patients' medical records by two independent investigators.

Genomic alteration concordance between ctDNA and tissue was determined using concordance rate and Kappa value with standard error (SE) for the three most commonly altered genes. Kappa value can range from 0 (rate of agreement expected by chance alone) to 1 (perfect agreement). Patients were stratified by time interval from tissue biopsy to ctDNA blood draw (≤ 6 months vs > 6 months) and tissue biopsy site (primary tumor vs metastatic site). Fisher's exact test was used to compare concordance rates.

Overall survival (OS) was determined from date of blood draw for first ctDNA to date of death or last follow‐up. Patients still alive at last follow‐up were censored on that date. Univariate analysis was performed to calculate hazard ratios (HR) for age, BMI, site of primary tumor, *TP53* alteration, *PIK3CA* alteration, median maximum ctDNA mutation allele frequency (MAF), number of characterized alterations, and number of lines of chemotherapy prior to first ctDNA collection; in general, cohorts were divided at the median of each value. All variables with *P* < 0.10 were included in the multivariate analysis. A second survival analysis was calculated in all patients who received matched treatment to ctDNA or unmatched treatment by either ctDNA or tissue, excluding patients who did not receive treatment or who received treatment matched only by tissue‐based molecular testing. This survival was determined from date of first matched treatment or date of unmatched treatment to date of death or last follow‐up. We added number of lines of chemotherapy prior to treatment to account for those patients who received matched treatment in greater than one subsequent line after ctDNA analysis.

## 3 Results

### 3.1 Patient characteristics

**Fig. 1:**
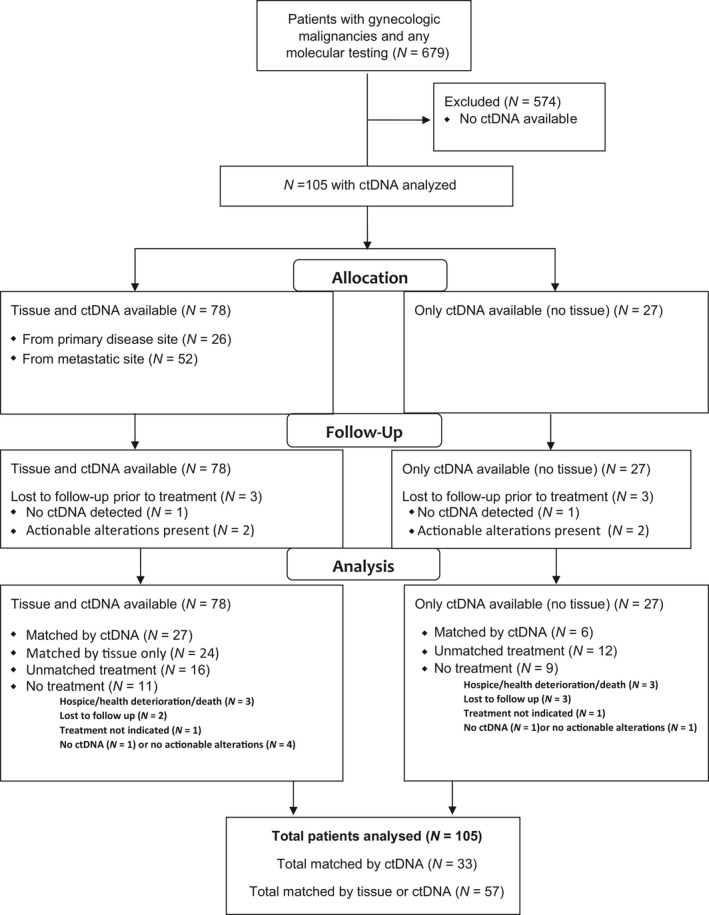
Consort diagram. 105 patients with ctDNA and 78 (74.3%) patients with accompanying tissue DNA sequencing. 33 (31.4%) matched by ctDNA. 24 (23.8%) matched by tissue DNA sequencing alone. Total number matched 57 (54.3%).

A total of 105 gynecologic cancer patients had ctDNA testing, and 78 patients (74.3%) had accompanying tissue tumor sequencing (Fig. 1). The median age was 64.1 years (range 31.5–80.8 years) at time of ctDNA analysis. The majority of gynecologic cancers were ovarian (*N* = 50, 47.6%), uterine (*N* = 37, 35.2%), or cervical (*N* = 13, 12.4%), with 80% of ovarian and 32% of uterine cancers high‐grade serous histology (Table 1). The majority of women were Caucasian (*N* = 89, 84.8%), and patients were treated with a median of two (range 0–13) lines of therapy prior to ctDNA testing (Table 1).

**Table 1:** Characteristics of patients with gynecologic cancers (*N* = 105).

Parameters	*N* (%)
Median age at diagnosis (range)	64.1 years (31.5–80.8 years)
Median BMI (range)	23 kg·m^−2^ (14–52 kg·m^−2^)
Ethnicity	
Caucasian	89 (84.8%)
Hispanic	8 (7.6%)
Middle Eastern	3 (2.9%)
Asian	2 (1.9%)
Black	2 (1.9%)
Other/Unknown	1 (1.0%)
Smoking status	
Never	74 (70.5%)
Former	25 (23.8%)
Current	3 (2.9%)
Unknown	3 (2.9%)
Histology by primary disease site	
Ovarian, fallopian tube, or primary peritoneal	50 (47.6%)
High‐grade serous carcinoma	40 (38.1%)
Clear cell carcinoma	3 (2.9%)
Low grade serous carcinoma	2 (1.9%)
Endometrioid adenocarcinoma	1 (1.0%)
Granulosa cell	1 (1.0%)
High‐grade carcinoma with neuroendocrine differentiation	1 (1.0%)
Poorly differentiated small cell carcinoma	1 (1.0%)
High‐grade transitional cell carcinoma	1 (1.0%)
*BRCA* alteration	17 (34.0% of ovarian cancers)
Detected on germline testing, negative ctDNA and tissue	7 (14.0% of ovarian cancers)
Detected on ctDNA, negative on tissue	1 (2.0% of ovarian cancers)
Detected on tissue, negative on ctDNA	8 (16.0% of ovarian cancers)
Detected on tissue and ctDNA	1 (2.0% of ovarian cancers)
Platinum sensitive at time of ctDNA collection	11 (22.0% of ovarian cancers)
Platinum resistant at time of ctDNA collection	39 (78.0% of ovarian cancers)
Uterine	37 (35.2%)
Serous carcinoma	12 (11.4%)
Endometrioid adenocarcinoma	
Grade 1	4 (3.8%)
Grade 2	5 (4.8%)
Grade 3	6 (5.7%)
Unknown grade	1 (1.0%)
Carcinosarcoma	4 (3.8%)
Sarcoma	2 (1.9%)
Clear cell carcinoma	1 (1.0%)
Perivascular epithelioid cell neoplasm (PEComa)	1 (1.0%)
High‐grade carcinoma with neuroendocrine differentiation	1 (1.0%)
Cervical, vulvar, and vaginal	13 cervical (12.4%), 2 vulvar (1.9%), and 1 vaginal (1.0%)
Squamous cell carcinoma	5 cervical (4.8%) + 2 vulvar (1.9%)
Adenocarcinoma	4 cervical (3.8%) + 1 vaginal (1.0%)
Adenosquamous carcinoma	2 (1.9%)
Neuroendocrine	1 (1.0%)
Clear cell carcinoma	1 (1.0%)
Unknown primary, presumed gynecologic	2 (1.9%)
Poorly differentiated carcinoma	2 (1.9%)
Median number of lines of therapy at time of ctDNA (range)	2 (0–13)
Median number of unique drugs received prior to ctDNA testing (range)	3 (0–15)
Neoadjuvant chemotherapy as part of initial treatment	9 (8.6%)
Primary surgery as part of initial treatment	88 (83.8%)
Secondary cytoreductive surgery during course of treatment	22 (21.0%)

### 3.2 ctDNA genomic characteristics

Of all patients, 79 (75.2%) had ≥ 1 characterized alteration in ctDNA (Table 2). The median number of ctDNA genomic alterations was one (range 0–13). The median maximum MAF, or highest percentage of any characterized alteration, of ctDNA per sample was 0.60% (range 0 to 75.6%). One patient (1.0%) was tested using the 54‐gene ctDNA panel, four patients (3.8%) with the 68‐gene panel, 35 patients (33.3%) with the 70‐gene panel, and 65 with patients (61.9%) the 73‐gene ctDNA panel (Table S1).

**Table 2:** DNA alterations and matching therapy (*N* = 105 patients).

	*N* (%)
Number of patients with ≥ 1 characterized ctDNA alteration	79 (75.2%)
Number of patients with accompanying tissue‐based sequencing	78 (74.3%)
Number of patients with no characterized ctDNA alterations (Includes patients with VUS only and those with no ctDNA detected)	26 (24.8%)
Number of patients with no characterized ctDNA alterations who had tissue sequencing done (*N* = 22) and showed ≥ 1 characterized alteration	22 (21.0%)
Number of ctDNA genomic alterations (median and range)^a^	1 (0–13)
Alteration with the highest percentage of tumor‐derived ctDNA (median and range)^a^ (Percentage of tumor‐derived cell‐free circulating DNA in comparison to wild‐type cell‐free DNA fragments at the same nucleotide position per sample)	0.60% (0–75.6%)
≥ 1 ctDNA alteration actionable by UCSD PREDICT criteria (13)	79 (75.2%)
≥ 1 ctDNA alteration actionable by OncoKB criteria (12)	55 (52.4%)
Matched to therapy by ctDNA	33 (31.4%)
Matched to therapy only by tissue‐based molecular alterations	24 (22.8%)
Total number of patients matched to therapy (ctDNA and/or tissue‐based testing)	57 (54.3%)
Primary reason for not matching to therapy by ctDNA (*N* = 72)	
No ctDNA detected on sample^b^	14 (13.3%)
Preferentially matched by tissue‐based molecular profile	12 (11.4%)
No actionable alteration^b^	12 (21.9%)
Received (unmatched) immunotherapy	8 (7.6%)
Received standard cytotoxic therapy	7 (6.7%)
Enrolled on a secondary unmatched clinical trial	6 (5.7%)
Hospice/health deterioration/death	6 (5.7%)
Lost to follow‐up	5 (4.8%)
Treatment not indicated at this time (patient doing well)	2 (1.9%)
Insurance issues	0 (0.0%)

^a^ Excluding VUS and synonymous alterations

^b^ If no ctDNA detected or no actionable alteration detected on ctDNA, this was coded as the primary reason for not matching by ctDNA. However, 12 of these patients were matched by alterations on tissue‐based molecular profiling, for a total of 24 patients matching by tissue molecular profiling alone.

**Fig. 2:**
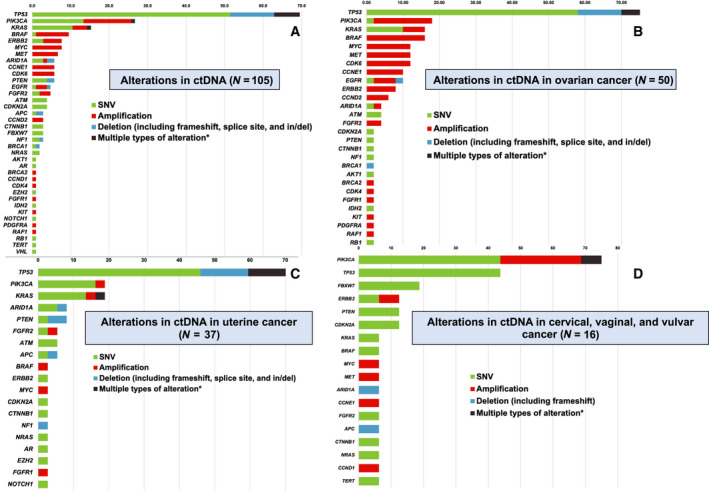
Frequency (% of patients) of characterized alterations in ctDNA (*N* = 105). Percentage of unique patients with alteration in each gene is shown after each bar.* (A) Frequency (% of patients) of characterized alterations in ctDNA in the gynecologic cancer cohort (*N* = 105). A total of 37 genes were altered in ctDNA analysis, with a total of 217 alterations in the 105 gynecologic cancer patients. (B) Frequency (% of patients) of characterized alterations in ctDNA in ovarian cancer cohort (*N* = 50). A total of 29 genes were altered in ctDNA analysis. (C) Frequency (% of patients) of characterized alterations in ctDNA in uterine cancer cohort (*N* = 37). A total of 19 genes were altered in ctDNA analysis. (D) Frequency (% of patients) of characterized alterations in ctDNA in cervical, vaginal, and vulvar cancer cohort (*N* = 16). A total of 18 genes were altered in ctDNA analysis. *Also shown in black bar is percentage of patients who had multiple types of alterations in the same gene. For example, if a patient had *PIK3CA* E545K SNV and *PIK3CA* amplification, each alteration was included in the bar graph under the SNV and the amplification categories and also in the category for multiple alterations. See also Table S1. Del, deletion; in/del, insertion/deletion.

In the 105 ctDNA samples, there were 217 unique genomic alterations. Gynecologic cancer patients most commonly had *TP53* (*N* = 59, 56.2%), *PIK3CA* (*N* = 26, 24.8%), *KRAS* (*N* = 14, 13.3%), *BRAF* (*N* = 10, 9.5%), *ERBB2* (*N* = 8, 7.6%), and *MYC* (*N* = 8, 7.6%) alterations (Fig. 2A, Table S2). Amplifications were more common in the ovarian cancer cohort when compared to uterine or cervical cancer cohorts (Fig. 2B–D). Ovarian cancer patients most commonly had alterations in *TP53* (*N* = 32, 64.0%), *PIK3CA* (*N* = 9, 18.0%), *KRAS* (*N* = 8, 16.0%)*, BRAF* (*N* = 8, 16.0%)*, MYC* (*N* = 6, 12.0%), *MET* (*N* = 6, 12.0%)*, CDK6* (*N* = 6, 12.0%)*, CCNE1* (*N* = 5, 10.0%), and *EGFR* (*N* = 5, 10.0%; Fig. 2B). Uterine cancer patients most commonly had alterations in *TP53* (*N* = 18, 48.6%), *PIK3CA* (*N* = 7, 18.9*%), KRAS* (*N* = 5, 13.5%), *ARID1A* (*N* = 3, 8.1%), and *PTEN* (*N* = 3, 8.1%; Fig. 2C)*. PIK3CA* (*N* = 8, 61.5%)*, TP53* (*N* = 5, 38.5%)*, FBXW7* (*N* = 3, 23.1%)*, ERBB2* (*N* = 2, 15.4%), and *PTEN* (*N* = 2, 15.4%) were most common in cervical cancer patients. Overall, 79 patients (75.2%) had ctDNA alterations that were potentially targetable by UCSD PREDICT criteria, while 55 women (52.4%) had ctDNA alterations that were actionable by OncoKB criteria [12, 13].

### 3.3 Higher maximum ctDNA mutation allelic frequency was associated with poor survival

Survival analysis included 105 patients, and the median time from ctDNA analysis to death or last follow‐up was 8 months. In univariate analysis, older age (≥ 64 years) and lower percentage MAF (< 0.6%) were significantly associated with improved OS, while fewer than three lines of prior chemotherapy showed a trend toward improved survival (Table 3). In multivariate analysis, older age [HR: 0.43, 95% confidence interval (CI): 0.25–0.75] and higher MAF (HR: 1.91, 95% CI: 1.08–3.38) (Fig. S1) remained independent prognostic factors for worse OS in gynecologic cancer patients (Table 3).

**Table 3:** Multivariate analysis of prognostic factors associated with OS from date of first ctDNA analysis in all patients with gynecologic malignancies (*N* = 105 patients, top) and in patients who were treated with matched therapies to ctDNA results or with unmatched treatment to either ctDNA or tissue (*N* = 61, bottom). NR, not reached; %ctDNA, mutant allele frequency.^a^

	Univariate analysis		Multivariate analysis^b^	
	Median OS (months)	*P*‐value	HR (95%CI)	*P*‐value
Characteristics (*N* = 105)				
Age at ctDNA analysis (years)				
≥ 64 (*N* = 53) vs < 64 (*N* = 52)	23.2 vs 7.3	0.009	0.43 (0.25–0.75)	0.003
BMI				
≥ 25 kg·m^−2^ (*N* = 45) vs < 25 kg·m^−2^ (*N* = 60)	23.0 vs 11.6	0.16	–	–
Site of primary tumor				
Ovary (*N* = 50) vs not (*N* = 55)	15.5 vs 23.0	0.93	–	–
Uterus (*N* = 37) vs not (*N* = 68)	23.0 vs 13.2	0.43	–	–
Cervix/Vulva/Vagina (*N* = 16) vs not (*N* = 89)	8.6 vs 20.0	0.22	–	–
Genomic alterations in ctDNA				
*TP53* (*N* = 59) vs not (*N* = 46)	11.6 vs 23.2	0.22	–	–
*PIK3CA* (*N* = 26) vs not (*N* = 79)	14.1 vs 23.0	0.14	–	–
Maximum MAF^c^				
≥ 0.6% (*N* = 53) vs < 0.6% (*N* = 52)	14.1 vs 23.2	0.06	1.91 (1.08–3.38)	0.03
Number of characterized alterations				
≥ 1 (*N* = 79) vs none (*N* = 26)	13.2 vs 23.2	0.35	–	–
Number of lines of chemotherapy prior to ctDNA analysis				
≥ 3rd line (*N* = 45) vs 1st or 2nd line (*N* = 60)	13.2 vs 23.2	0.06	1.70 (0.99–2.92)	0.06
Analysis limited to patients treated with matched therapies to ctDNA results or patients with unmatched treatment to either ctDNA or tissue^a^				
Characteristics (*N* = 61)				
Age at ctDNA analysis (years)				
≥ 64 (*N* = 31) vs < 64 (*N* = 30)	25.8 vs 5.5	0.02	0.44 (0.22–0.90)	0.02
BMI				
≥ 25 kg·m^−2^ (*N* = 28) vs < 25 kg·m^−2^ (*N* = 33)	20.0 vs 6.6	0.16	–	–
Site of primary tumor				
Ovary (*N* = 26) vs not (*N* = 35)	15.5 vs 14.1	0.81	–	–
Uterus (*N* = 22) vs not (*N* = 39)	23.0 vs 13.2	0.69	–	–
Cervix/Vulva/Vagina (*N* = 11) vs not (*N* = 50)	8.6 vs 15.5	0.41	–	–
Genomic alterations in ctDNA				
*TP53* (*N* = 41) vs not (*N* = 20)	13.2 vs NR	0.61	–	–
*PIK3CA* (*N* = 20) vs not (*N* = 41)	14.1 vs 13.2	0.74	–	–
Maximum MAF^c^				
≥ 0.6% (*N* = 37) vs < 0.6% (*N* = 24)	15.5 vs 13.2	0.69	–	–
Number of characterized alterations				
≥ 1 (*N* = 54) vs none (*N* = 7)	14.1 vs 4.0	0.55	–	–
Treatment following ctDNA analysis^d^				
Matched by ctDNA (*N* = 33) vs unmatched by either ctDNA or tissue (*N* = 28)	20.0 vs 5.3	0.005	0.34 (0.16–0.75)	0.007
Number of lines of treatment before the matched or unmatched therapy				
≥ 3rd line (*N* = 26) vs 1st or 2nd line (*N* = 35)	7.2 vs 15.5	0.21	–	–

^a^ Patients who were treated with therapies matching to tissue‐based DNA results only were excluded from the second analysis. Survival in first analysis (top) was calculated from date of first ctDNA to date of last follow‐up or death. Survival in second analysis (bottom) was calculated from start of treatment: first matched treatment after ctDNA in the matched group or the first treatment after ctDNA in the unmatched group

^b^ Factors with *P*‐value < 0.1 in univariate analysis were included in the multivariate analysis

^c^ Only characterized alterations were considered (synonymous alterations and VUS were excluded). Dichotomized at the median of 0.6% for the maximum percentage mean allelic frequency ctDNA per sample.

^d^ Only treated patients included.

### 3.4 Therapy matched to ctDNA genomic alterations was independently associated with improved overall survival

Thirty‐three patients (31.4%) were matched by ctDNA. An additional 24 patients (22.8%) were matched by tissue‐based molecular alterations, with a total of 57 patients (54.3%) matching to targeted therapy by molecular analysis. Seventy‐two patients did not match to treatment by ctDNA, with reasons outlined in Table 2. Notably, the most common indications for not pursuing ctDNA‐matched therapy were no ctDNA detected (*N* = 14, 19.4%), no actionable alterations detected (*N* = 12, 16.6%), and preferentially matched by tissue‐based molecular profile (*N* = 12, 16.6%). Importantly, no patient failed to match to targeted therapy due to financial issues (Table 2). When the survival analysis was restricted to the 33 patients who received treatment matched by ctDNA and the 28 patients who received unmatched (either by ctDNA or tissue‐based molecular analysis) treatment, older age (HR: 0.44, 95% CI: 0.22–0.90) and matched treatment (HR: 0.34, 95% CI: 0.16–0.75) remained independent prognostic factors for OS in the multivariate analysis (Table 3, Fig. S1).

### 3.5 Tissue and ctDNA genomic results showed high concordance unaffected by temporal or spatial factors

Of the 105 gynecologic cancer patients with ctDNA testing, 78 patients (74.3%) had accompanying tissue‐based molecular testing. One patient (1.3%) had completely concordant, and 42 patients (53.8%) had partially concordant results between ctDNA and tissue‐based molecular testing. The concordance rate was 75.6% (Kappa: 0.51, SE: 0.10) for *TP53*, 78.2% (Kappa: 0.42, SE: 0.12) for *PIK3CA*, and 88.5% (Kappa: 0.60, SE 0.12) for *KRAS* (Table 4). Concordance was not significantly correlated with location of tissue biopsy (primary vs metastatic site) or time interval between blood draw and tissue biopsy (Table 4).

**Table 4:** Overall concordance between ctDNA and tissue‐based DNA by tissue biopsy site (primary or metastatic) and time interval between blood draw and tissue biopsy (*N* = 78).

Patients who had both ctDNA and tissue DNA sequencing (*N* = 78)^a^					
		Tissue DNA (+)	Tissue DNA (−)	Overall concordance	Kappa^b^ (SE)
*TP53*	ctDNA (+)	35	6	75.6%	0.51 (0.10)
	ctDNA (−)	13	24		
*PIK3CA*	ctDNA (+)	11	6	78.2%	0.42 (0.12)
	ctDNA (−)	11	50		
*KRAS*	ctDNA (+)	9	3	88.5%	0.60 (0.12)
	ctDNA (−)	6	60		

Concordance based on whether primary tumor or metastatic site was biopsied									
	Primary tumor (*N* = 26)				Metastatic sites (*N* = 52)				*P*‐value (primary tumor vs metastatic sites)
	(+/+)	(−/−)	Overall concordance	Kappa (SE)	(+/+)	(−/−)	Overall concordance	Kappa (SE)	
*TP53*	*N* = 11	*N* = 11	84.6%	0.69 (0.14)	*N* = 24	*N* = 13	71.2%	0.41 (0.12)	0.27
*PIK3CA*	*N* = 6	*N* = 12	69.2%	0.37 (0.18)	*N* = 5	*N* = 38	82.7%	0.42 (0.16)	0.25
*KRAS*	*N* = 3	*N* = 20	88.5%	0.60 (0.21)	*N* = 6	*N* = 40	88.5%	0.60 (0.15)	>0.99

Concordance based on time interval between blood draw and tissue biopsy									
	≤ 6 months (*N* = 32)				>6 months (*N* = 46)				*P*‐value (≤ 6 vs > 6 months)
	(+/+)	(−/−)	Overall concordance	Kappa (SE)	(+/+)	(−/−)	Overall concordance	Kappa (SE)	
*TP53*	*N* = 16	*N* = 10	81.3%	0.61 (0.14)	*N* = 19	*N* = 14	71.7%	0.44 (0.13)	0.43
*PIK3CA*	*N* = 5	*N* = 19	75.0%	0.39 (0.17)	*N* = 6	*N* = 31	80.4%	0.45 (0.16)	0.59
*KRAS*	*N* = 1	*N* = 28	90.6%	0.37 (0.27)	*N* = 8	*N* = 32	87.0%	0.64 (0.13)	0.73

^a^ Genomically concordant (e.g., if a patient had *KRAS* amplification in ctDNA and *KRAS* G12S in tissue DNA, counted as ‘concordant’)

^b^ Kappa value can range from 0 (rate of agreement expected by chance alone) to 1 (perfect agreement), with a higher kappa value correlating to a better concordance.

## 4 Discussion

Gynecologic malignancies diagnosed at an advanced stage often recur and are difficult to treat. Although prognosis varies by primary disease site, recurrent gynecologic cancer is generally incurable and treatment options exhibit modest efficacy with accompanying toxicity. The molecular characterization of gynecologic malignancies has emerged as an area of active interest; however, the utility of ctDNA to guide treatment in gynecologic cancer and its correlation with clinical data have been limited [14, 15].

We found that 75.2% of gynecologic cancer patients had ≥ 1 genomic alteration on ctDNA assessment (Table 1). *TP53* alterations were seen in over 50% of patients, and *PIK3CA* alterations were seen in nearly 25% of patients. These numbers are similar to a recent publication of 2579 ‘pan‐gynecologic cancer’ patients (1087 breast cancers, 579 ovarian cancers, 548 endometrial cancers, 308 cervical cancers, and 57 uterine carcinosarcomas) in The Cancer Genome Atlas; they reported *TP53* and *PIK3CA* alteration rates of 44% and 32%, respectively [14]. However, their cohort had higher rates of *PTEN* (20% vs 5.7%) and *ARID1A* (14% vs 5.7%) alterations than our cohort.

We considered 100% of the characterized alterations to be targetable by FDA‐approved agents or therapies in development, while 71% of characterized alterations were considered targetable by OncoKB criteria [12, 13]. This discrepancy is likely explained by UCSD PREDICT criteria defining *TP53* as targetable using antiangiogenic agents based on prior data, while OncoKB has not defined this relationship; multiple studies have now demonstrated that TP53 is a marker for increased VEGF expression and improved response to antiangiogenic agents [16, 17, 18, 19, 20]. This percentage is higher than that described in a prior report of 211 gynecologic cancer patients, where 48% had at least one actionable alteration or a recent study of 78 high‐grade serous ovarian cancer ctDNA samples that showed 58% had at least one actionable [21]. Regardless, these observations suggest that many gynecologic cancer patients may be candidates for matched treatment [9, 21, 22, 23, 24, 25, 26]. Furthermore, these data indicate discerning druggable alterations can be achieved through ctDNA analysis, which is less invasive, more convenient, and may afford more contemporaneous samples than tissue biopsy. Similar to reports of ctDNA examination in other cancer patients, our gynecologic cohort mostly had unique genomic portfolios in ctDNA, emphasizing the opportunity for individualized therapy [27, 28, 29, 30].

The overall concordance rate of genomic alterations between tissue and ctDNA was 75.6–88.5% for *TP53*, *PIK3CA*, and *KRAS*. Concordance rates were not significantly related to location of biopsy (primary vs metastatic site) or time interval between blood draw and tissue biopsy (Table 4). These concordance rates provide some reassurance for reliability of ctDNA in place of tissue biopsy; however, tissue biopsy may add more actionable targets than ctDNA alone, as tissue‐based NGS panels often comprise a much larger targeted set of genes.

Similar to prior studies in a variety of nongynecologic cancers, we found that higher percentage of ctDNA was correlated with worse survival [27, 30, 31, 32, 33]. ctDNA has recently been associated with increased risk of recurrence in colorectal cancer and poorer outcomes in advanced non‐small‐cell lung carcinoma, breast cancer, and ovarian cancer [34, 35, 36, 37]. In 44 patients with ovarian or uterine serous cancers who completed frontline therapy, Pereira *et al*. showed that ctDNA can be used as a biomarker to predict disease persistence and recurrence and was associated with OS [38]. Somewhat surprisingly, in our cohort, younger age was significantly associated with poorer OS. This may be due to selection bias, as our younger patients may have been more likely to be referred to our precision medicine program or offered ctDNA despite poorer performance status or more advanced malignancies. Alternatively, being a tertiary care center, it is conceivable that there is a referral bias for young patients with more aggressive disease.

Matched therapy has previously been shown to have great promise in cancer therapy [3, 24, 28, 29, 39, 40]. We demonstrate that matched therapy by ctDNA was associated with significant improvement in OS in univariate and multivariate analysis, with 20.0‐month median OS in the matched cohort compared to 5.3 months in the patients who received unmatched treatment. This demonstrates that ctDNA may be used to direct therapy to improve OS in gynecologic cancer. However, because patients were treated with heterogeneous matched and unmatched treatments (Table S3), further study is warranted to definitively conclude that matched therapy to ctDNA improves survival in gynecologic cancer.

This study has some important limitations. For simplicity, we considered only each patient's first ctDNA sample in our analyses. It is possible that patients had subsequent ctDNA analyses that were used for matched therapy; however, these matches were not used in our analysis. Additionally, our cohort is relatively small with a mix of ovarian, uterine, and cervical cancers, and all patients were treated at a single institution; however, in univariate analysis, disease site was not associated with OS, so it was unlikely to be a confounder. Furthermore, the vast majority of our tumors were high grade, and therefore, the impact of grade on our findings could not be elucidated. Similarly, only 22% (*N* = 11) of the 40 ovarian cancers were platinum sensitive, reflecting this heavily pretreated population. Thirty‐four percent (*N* = 17) of ovarian cancer patients had *BRCA* alterations detected on germline or somatic tissue or ctDNA testing, while only 4% (*N* = 2) had *BRCA* alterations detected on ctDNA (Table 1). Of note, Guardant does not report germline alterations on ctDNA, which is an important limitation of this test, and explains the much of the discrepancy in these numbers. Also, ctDNA may have missed some somatic alterations captured by tissue biopsy due to relatively lower disease burden, including three patients with maximal mean allele frequency < 2.0%. Of note, the *BRCA*‐positive patient with the highest maximum allele frequency (48.1%) did have her *BRCA* alteration detected on ctDNA. We advocate for further study into the important issue of concordance of *BRCA* alterations on ctDNA with tissue biopsy and recommend combining ctDNA with germline and/or tissue testing to definitively rule out *BRCA* alterations, especially given its significant clinical relevance for predicted benefit from PARP inhibitors [4]. The small numbers of patients in each of these subsets rendered it difficult to assess these important variables, which should be evaluated in follow‐up studies of larger numbers of patients. Also, not all patients with ctDNA testing also had tissue‐based testing, potentially limiting our concordance analyses. Finally, although we found that patients matched to ctDNA had improved OS, we did not examine progression‐free survival or response rates due to heterogeneous patient follow‐up; it is therefore conceivable that OS could be confounded by subsequent treatment after matched therapy. Despite these limitations, our findings are important in informing the utility of ctDNA in the treatment of gynecologic cancers.

Importantly, financial barriers did not impact access to targeted therapy in this patient cohort, despite diverse socioeconomic backgrounds. This likely reflects implementation of a medication acquisition team as part of our precision medicine program, as well as a robust portfolio of clinical trials and the availability of clinical trial coordinators [28]. As shown in other cancer types, ctDNA has great potential as an important biomarker to predict response to immunotherapy, guide need for adjuvant therapy in the postoperative setting, monitor response to therapy, and predict resistance or recurrences months prior to imaging [31, 41, 42, 43]. Further study and validation are required for these exciting potential future uses.

## 5 Conclusions

Efforts to improve oncologic outcomes and treatment options for patients with gynecologic cancers remain a clinical priority. This study suggests that ctDNA assessment may have both prognostic and therapeutic implications, informing individualized cancer therapy in a cohort of patients with gynecologic cancer. We found that higher ctDNA maximum MAF was associated with worse OS, while therapy matched to ctDNA genomic alterations was independently associated with improved OS compared to unmatched therapy. Tissue and ctDNA genomic results showed high concordance unaffected by temporal or spatial factors. Additional studies are warranted to better define the utility of ctDNA assessment in the management of gynecologic cancer.

## 7 Conflicts of interest

RNE—is consultant in Tesaro, Clovis Oncology, AstraZeneca Pfizer, and Merck; received honoraria from AstraZeneca, Clovis Oncology, and Genentech; provided institutional research funding to Genentech‐Roche and Merck. SPP—is consultant in AstraZeneca, Bristol‐Myers Squibb, Eli Lilly, Illumina, Nektar, Novartis, and Tempus and provided institutional research funding to Bristol‐Myers Squibb, Eli Lilly, Fate, Incyte, AstraZeneca/MedImmune, Merck, Pfizer, Roche/Genentech, Xcovery, Fate Therapeutics, Genocea, and Iovance. RL—is employee and stock owner in Guardant Health; is board member in Biolase, Inc.; and is advisor with stock ownership in Forward Medical, Inc.. DEP—is consultant in Tocagen. SK—is consultant in Foundation Medicine and received honoraria from Roche. MTM—is consultant in Tesaro and Eisai. RK—provided research funding to Incyte, Genentech, Merck Serono, Pfizer, Sequenom, Foundation Medicine, Guardant Health, Grifols, and Konica Minolt; consultant in LOXO, X‐Biotech, Actuate Therapeutics, Genentech and NeoMed; received speaker fees from Roche; and provided ownership interest in IDbyDNA and Curematch, Inc..

## 9 Author contributions

LMC, RNE, and RK designed the study. LMC and RO abstracted and analyzed the data. LMC wrote the manuscript, and RNE, RO, SPP, MN, RBL, DEP, SK, MTM, and RK substantively revised it. All authors read and approved the final manuscript.

## Supporting information


**Fig. S1.** Kaplan Meier survival curves for OS.
**Table S1.** 54‐ to 73‐gene panels (Guardant, Inc.)
**Table S2.** Alterations in gynecologic patients undergoing ctDNA testing (*N* = 105 patients)*.
**Table S3.** Patient‐level DNA alterations and therapy in patients who received treatment after ctDNA (*N* = 85 patients).

## Data Availability

The data are not available in a public database or repository. The datasets generated and analyzed in this study are available from the corresponding author on reasonable request.
