# Supplementary material for: Clinical implications of plasma circulating tumor DNA in gynecologic cancer patients
Source: Mol Oncol. 2020 Sep 17;15(1):67–79. doi: 10.1002/1878-0261.12791 (PMC7782073; doi:10.1002/1878-0261.12791)
Supplement: Supplementary file 1 — Fig. S1. Kaplan Meier survival curves for OS. Table S1. 54‐ to 73‐gene panels (Guardant, Inc.) Table S2. Alterations in gynecologic patients undergoing ctDNA testing (N = 105 patients)*. Table S3. Patient‐level DNA alterations and therapy in patients who received treatment after ctDNA (N = 85 patients). [file MOL2-15-67-s001.docx]

**SUPPLEMENTARY MATERIALS**

**Table S1.** 54- to 73-gene panels (*Guardant, Inc*.).

**Supplemental Table 1a.** 54-gene panel

| **POINT MUTATIONS** | | | | **AMPLIFICATIONS** |
| --- | --- | --- | --- | --- |
| *ABL1* | *AKT1* | ***ALK*** | ***APC*** | *EGFR* |
| ***AR*** | *ATM* | ***BRAF*** | ***CDKN2A*** | *ERBB2* |
| *CDH1* | *CSF1R* | *CTNNB1* | ***EGFR*** | *MET* |
| ***ERBB2*** | *ERBB4* | *EZH2* | ***FBXW7*** |  |
| *FGFR1* | *FGFR2* | *FGFR3* | *FLT3* |  |
| *GNA11* | *GNAQ* | *GNAS* | *HNF1A* |  |
| *HRAS* | *IDH1* | *IDH2* | *JAK2* |  |
| *JAK3* | *KDR* | *KIT* | ***KRAS*** |  |
| ***MET*** | *MLH1* | *MPL* | ***MYC*** |  |
| ***NOTCH1*** | *NPM1* | ***NRAS*** | *PDGFRA* |  |
| ***PIK3CA*** | ***PTEN*** | *PTPN11* | ***PROC*** |  |
| ***RB1*** | *RET* | *SMAD4* | *SMARCB1* |  |
| *SMO* | *SRC* | *STK11* | *TERT* |  |
| ***TP53*** | *VHL* |  |  |  |

All exons were sequenced in genes in ***bold***.

**Supplemental Table 1b.** 68-gene panel

| **POINT MUTATIONS** | | | | **AMPLIFICATIONS** | **FUSIONS** | **INDELS** |
| --- | --- | --- | --- | --- | --- | --- |
| *AKT1* | *ALK* | ***APC*** | ***AR*** | *AR* | *ALK* | *EGFR* exon 19 deletions |
| *AFAR* | ***ARID1A*** | *ATM* | ***BRAF*** | *BRAF* | *NTRK1* | *EGFR* exon 20 insertions |
| ***BRCA1*** | ***BRCA2*** | ***CCDN1*** | ***CCDN2*** | *CCNE1* | *RET* |  |
| ***CCNE1*** | *CDH1* | ***CDK4*** | ***CDK6*** | *CDK4* | *ROS1* |  |
| ***CDKN2A*** | ***CDKN2B*** | *CTNNB1* | ***EGFR*** | *CDK6* |  |  |
| ***ERBB2*** | *ESR1* | *EZH2* | *FBXW7* | *EGFR* |  |  |
| ***FGFR1*** | ***FGFR2*** | *FGFR3* | *GATA3* | *ERBB2* |  |  |
| *GNA11* | *GNAQ* | *GNAS* | *HNF1A* | *FGFR1* |  |  |
| ***HRAS*** | *IDH1* | *IDH2* | *JAK2* | *FGFR2* |  |  |
| *JAK3* | ***KIT*** | ***KRAS*** | *MAP2K1* | *KIT* |  |  |
| *MAP2K2* | ***MET*** | *MLH1* | *MPL* | *KRAS* |  |  |
| ***MYC*** | ***NF1*** | *NFE2L2* | *NOTCH1* | *MET* |  |  |
| *NPM1* | ***NRAS*** | *NTRK1* | ***PDGFRA*** | *MYC* |  |  |
| ***PIK3CA*** | ***PTEN*** | *PTPN11* | ***RAF1*** | *PDGFRA* |  |  |
| *RET* | *RHEB* | *RHOA* | *RIT1* | *PIK3CA* |  |  |
| *ROS1* | *SMAD4* | *SMO* | *SRC* | *RAF1* |  |  |
| *STK11* | *TERT* | ***TP53*** | *VHL* |  |  |  |

Complete exon coverage for genes in ***bold***.

**Supplemental Table 1c.** 70-gene panel

| **POINT MUTATIONS** | | | | **AMPLIFICATIONS** | **FUSIONS** | **INDELS** |
| --- | --- | --- | --- | --- | --- | --- |
| *AKT1* | *ALK** | ***APC*** | ***AR*** | *AR* | *ALK* | *EGFR* exon 19 deletions |
| *ARAF* | ***ARID1A*** | *ATM* | ***BRAF*** | *BRAF* | *FGFR2* | *EGFR* exon 20 insertions |
| ***BRCA1*** | ***BRCA2*** | ***CCND1*** | ***CCND2*** | *CCND1* | *FGFR3* | *ERBB2* exon 19 deletions |
| ***CCNE1*** | *CDH1* | ***CDK4*** | ***CDK6*** | *CCND2* | *NTRK1* | *ERBB2* exon 20 insertions |
| ***CDKN2A*** | ***CDKN2B*** | *CTNNB1* | ***EGFR*** | *CCNE1* | *RET* |  |
| ***ERBB2*** | *ESR1* | *EZH2* | *FBXW7* | *CDK4* | *ROS1* |  |
| ***FGFR1*** | ***FGFR2**** | *FGFR3** | *GATA3* | *CDK6* |  |  |
| *GNA11* | *GNAQ* | *GNAS* | *HNF1A* | *EGFR* |  |  |
| ***HRAS*** | *IDH1* | *IDH2* | *JAK2* | *ERBB2* |  |  |
| *JAK3* | ***KIT*** | ***KRAS*** | *MAP2K1* | *FGFR1* |  |  |
| *MAP2K2* | ***MET*** | *MLH1* | *MPL* | *FGFR2* |  |  |
| ***MYC*** | ***NF1*** | *NFE2L2* | *NOTCH1* | *KIT* |  |  |
| *NPM1* | ***NRAS*** | *NTRK1** | ***PDGFRA*** | *KRAS* |  |  |
| ***PIK3CA*** | ***PTEN*** | *PTPN11* | ***RAF1*** | *MET* |  |  |
| ***RB1*** | *RET** | *RHEB* | *RHOA* | *MYC* |  |  |
| *RIT1* | *ROS1** | *SMAD4* | *SMO* | *PDGFRA* |  |  |
| *SRC* | *STK11* | *TERT* | ***TP53*** | *PIK3CA* |  |  |
| *TSC1* | *VHL* |  |  | *RAF1* |  |  |

Complete exon and partial intron coverage for genes in ***bold***. *Genes with asterisk include rearrangements. *MET* includes exon 14 skipping.

**Supplemental Table 1d.** 73-gene panel

| **POINT MUTATIONS** | | | | **AMPLIFICATIONS** | **FUSIONS** | **INDELS** | |
| --- | --- | --- | --- | --- | --- | --- | --- |
| *AKT1* | *ALK* | *APC* | *AR* | *AR* | *ALK* | *APC* | *ARID1A* |
| *ARAF* | *ARID1A* | *ATM* | *BRAF* | *BRAF* | *FGFR2* | *ATM* | *BRCA1* |
| *BRCA1* | *BRCA2* | *CCND1* | *CCND2* | *CCND1* | *FGFR3* | *BRCA2* | *CDH1* |
| *CCNE1* | *CDH1* | *CDK4* | *CDK6* | *CCNE1* | *NTRK1* | *CDKN2A* | *EGFR* |
| *CDKN2A* | *CTNNB1* | *DDR2* | *EGFR* | *CDK4* | *RET* | *GATA3* | *KIT* |
| *ERBB2* | *ESR1* | *EZH2* | *FBXW7* | *CDK6* | *ROS1* | *MET* | *MLH1* |
| *FGFR1* | *FGFR2* | *FGFR3* | *GATA3* | *EGFR* |  | *MTOR* | *NF1* |
| *GNA11* | *GNAQ* | *GNAS* | *HNF1A* | *ERBB2* |  | *PDGFRA* | *PTEN* |
| *HRAS* | *IDH1* | *IDH2* | *JAK2* | *FGFR1* |  | *RB1* | *SMAD4* |
| *JAK3* | *KIT* | *KRAS* | *MAP2K1* | *FGFR2* |  | *STK11* | *TP53* |
| *MAP2K2* | *MAPK1* | *MAPK3* | *MET* | *KIT* |  | *TSC1* | *VHL* |
| *MLH1* | *MPL* | *MTOR* | *MYC* | *KRAS* |  |  |  |
| *NF1* | *NFE2L2* | *NOTCH1* | *NPM1* | *MET* |  |  |  |
| *NRAS* | *NTRK1* | *NTRK3* | *PDGFRA* | *MYC* |  |  |  |
| *PIK3CA* | *PTEN* | *PTPN11* | *RAF1* | *PDGFRA* |  |  |  |
| *RB1* | *RET* | *RHEB* | *RHOA* | *PIK3CA* |  |  |  |
| *RIT1* | *ROS1* | *SMAD4* | *SMO* | *RAF1* |  |  |  |
| *STK11* | *TERT* | *TP53* | *TSC1* |  |  |  |  |
| *VHL* |  |  |  |  |  |  |  |

*TERT* includes alterations in the promoter region. *MET* includes exon 14 skipping.

**Table S2:** Alterations in gynecologic patients undergoing ctDNA testing (N=105 patients)*

|  | SNV (%) | Amplification (%) | Insertion/Deletion (%) | Multiple types of alterations (%) | Total Alterations  (N=217) | Total Patients  (%) |
| --- | --- | --- | --- | --- | --- | --- |
| *TP53* | 54 (51.4) | 0 (0) | 12 (11.4) | 7 (6.7) | 66 | 59 (56.2) |
| *PIK3CA* | 14 (13.3) | 13 (12.4) | 0 (0) | 1 (1.0) | 27 | 26 (24.8) |
| *KRAS* | 11 (10.5) | 4 (3.8) | 0 (0) | 1 (1.0) | 15 | 14 (13.3) |
| *BRAF* | 1 (1.0) | 9 (7.6) | 0 (0) | 0 (0) | 10 | 10 (9.5) |
| *ERBB2* | 3 (2.9) | 5 (4.8) | 0 (0) | 0 (0) | 8 | 8 (7.6) |
| *MYC* | 0 (0) | 8 (7.6) | 0 (0) | 0 (0) | 8 | 8 (7.6) |
| *MET* | 0 (0) | 7 (7.6) | 0 (0) | 0 (0) | 7 | 7 (6.7) |
| *ARID1A* | 3 (2.9) | 1 (1.0) | 2 (1.9) | 0 (0) | 6 | 6 (5.7) |
| *CCNE1* | 0 (0) | 6 (5.7) | 0 (0) | 0 (0) | 6 | 6 (5.7) |
| *CDK6* | 0 (0) | 6 (5.7) | 0 (0) | 0 (0) | 6 | 6 (5.7) |
| *PTEN* | 4 (3.8) | 0 (0) | 2 (1.9) | 0 (0) | 6 | 6 (5.7) |
| *EGFR* | 1 (1.0) | 3 (2.9) | 1 (1.0) | 0 (0) | 5 | 5 (4.8) |
| *FGFR2* | 2 (1.9) | 3 (2.9) | 0 (0) | 0 (0) | 5 | 5 (4.8) |
| *ATM* | 4 (3.8) | 0 (0) | 0 (0) | 0 (0) | 4 | 4 (3.8) |
| *CDKN2A* | 4 (3.8) | 0 (0) | 0 (0) | 0 (0) | 4 | 4 (3.8) |
| *APC* | 1 (1.0) | 0 (0) | 2 (1.9) | 0 (0) | 3 | 3 (2.9) |
| *CCND2* | 0 (0) | 3 (2.9) | 0 (0) | 0 (0) | 3 | 3 (2.9) |
| *CTNNB1* | 3 (2.9) | 0 (0) | 0 (0) | 0 (0) | 3 | 3 (2.9) |
| *FBXW7* | 3 (2.9) | 0 (0) | 0 (0) | 0 (0) | 3 | 3 (2.9) |
| *NF1* | 2 (1.9) | 0 (0) | 1 (1.0) | 0 (0) | 3 | 3 (2.9) |
| *BRCA1* | 1 (1.0) | 0 (0) | 1 (1.0) | 0 (0) | 2 | 2 (1.9) |
| *NRAS* | 2 (1.9) | 0 (0) | 0 (0) | 0 (0) | 2 | 2 (1.9) |
| *AKT1* | 1 (1.0) | 0 (0) | 0 (0) | 0 (0) | 1 | 1 (1.0) |
| *AR* | 1 (1.0) | 0 (0) | 0 (0) | 0 (0) | 1 | 1 (1.0) |
| *BRCA2* | 0 (0) | 1 (1.0) | 0 (0) | 0 (0) | 1 | 1 (1.0) |
| *CCND1* | 0 (0) | 1 (1.0) | 0 (0) | 0 (0) | 1 | 1 (1.0) |
| *CDK4* | 0 (0) | 1 (1.0) | 0 (0) | 0 (0) | 1 | 1 (1.0) |
| *EZH2* | 1 (1.0) | 0 (0) | 0 (0) | 0 (0) | 1 | 1 (1.0) |
| *FGFR1* | 0 (0) | 1 (1.0) | 0 (0) | 0 (0) | 1 | 1 (1.0) |
| *IDH2* | 1 (1.0) | 0 (0) | 0 (0) | 0 (0) | 1 | 1 (1.0) |
| *KIT* | 0 (0) | 1 (1.0) | 0 (0) | 0 (0) | 1 | 1 (1.0) |
| *NOTCH1* | 1 (1.0) | 0 (0) | 0 (0) | 0 (0) | 1 | 1 (1.0) |
| *PDGFRA* | 0 (0) | 1 (1.0) | 0 (0) | 0 (0) | 1 | 1 (1.0) |
| *RAF1* | 0 (0) | 1 (1.0) | 0 (0) | 0 (0) | 1 | 1 (1.0) |
| *RB1* | 1 (1.0) | 0 (0) | 0 (0) | 0 (0) | 1 | 1 (1.0) |
| *TERT* | 1 (1.0) | 0 (0) | 0 (0) | 0 (0) | 1 | 1 (1.0) |
| *VHL* | 1 (1.0) | 0 (0) | 0 (0) | 0 (0) | 1 | 1 (1.0) |

*See also Figure 2. Numbers (percent) of patients with specific DNA alterations are shown. If a ctDNA sample had multiple alterations in the same gene of the same type, it was counted once. However, if a patient had different types of alterations within the same gene, each type of alteration was counted and this was noted in multiple types of alterations. For example, a patient with a *TP53* SNV and amplification had each alteration counted. However, a patient with a *TP53* C141Y SNV and a *TP53* R249G SNV had only one *TP53* SNV alteration counted.

**Figure S1:** Kaplan Meier survival curves for overall survival. **Figure 1a:** Overall survival in all patients with gynecologic malignancies (N=105 patients) by maximum mutation allele frequency (MAF), dichotomized at median of 0.6%. Overall survival was calculated from date of first ctDNA analysis to date of last follow up or death. **Figure 1b:** Overall survival in patients who were treated with matched therapies to ctDNA results or with unmatched treatment to either ctDNA or tissue (N=61).* Overall survival was calculated from start of treatment (first matched treatment after ctDNA in the matched group or the first treatment after ctDNA in the unmatched group) to last follow up or death.

* Patients who were treated with therapies matching to tissue-based DNA results only were excluded from the second analysis.

**Table S3: Patient-level DNA alterations and therapy in patients who received treatment after ctDNA (N=85 patients)**

| Study ID | OS (months) † | Characterized alterations in first ctDNA | Actionable by oncoKB^[13]^ | Actionable by UC San Diego^[14]^ | Characterized alterations in tissue | Treatment by matching†† | Treatment Drugs (targeted alteration) |
| --- | --- | --- | --- | --- | --- | --- | --- |
| 763 | 27.1 | ATM R3008H  TP53 Y236D | Yes  No | Yes  Yes | AURKA amplification  GNAS amplification  LYN amplification  PRKCI amplification  TERC amplification  TP53 R273H  ZNF217 amplification | Matched by ctDNA | Olaparib (ATM, BRIP1), bevacizumab (TP53)* |
| 1096 | 31.8 | None | No | No | AKT2 amplification  BRCA1 Y655fs*18  CCNE1 amplification – equivocal  MLL2 deletion exons 19-28  MYCN amplification – equivocal  NF1 splice site 587-2A>C  PRKCI amplification – equivocal  TERC amplification – equivocal  TP53 splice site 993+1G>A | Matched to tissue-based biopsy | Bevacizumab (TP53)* |
| 1215 | 50.4 | TP53 K132E | No | Yes | FAS K287*  TP53 I255S | Matched by ctDNA | Bevacizumab (TP53)* |
| 1262 | 23.0 | None | No | No | ARID1A F266*  PIK3R1 Q579fs*23  PIK3R1 splice site 1746-8_1752del15  PTEN R130*  PTEN R233* | Matched to tissue-based biopsy | Everolimus (PIK3CA, PTEN) |
| 1421 | 9.4 | None | No | No | CDKN2A/B loss | Matched to tissue-based biopsy | Palbociclib (CDKN2A) |
| 1693 | 11.1 | None | No | No | CHD4 R975H  IDH1 R132H | Unmatched by either ctDNA or tissue | Ifosfamide |
| 1948 | 5.0 | TP53 Splice Site  KRAS G12V  ATM R3008H | No  Yes  Yes | Yes  Yes  Yes | ATR E1602*  BRAF amplification – equivocal  CCND2 amplification  CD274 (PD-L1) amplification  FGF23 amplification  FGF6 amplification  JAK2 amplification – equivocal  KDM5A amplification  KEL amplification – equivocal  KRAS amplification – equivocal, G12V  MYC amplification  PDCD1LG2 (PD-L2) amplification  TP53 splice site 783-2A>C | Matched by ctDNA | Niraparib (ATM) |
| 1994 | 37.5 | None | No | No | BRCA1 Q1756fs*74  CIC F524fs*150  RB1 splice site 2483_2489+34del41  TP53 R249T | Matched to tissue-based biopsy | Carboplatin (BRCA1) |
| 1997 | 23.2 | None | No | No | CDKN2A p16INK4a R58*, p14ARFP72L  ZC3H7B-BCOR fusion | Matched to tissue-based biopsy | Palbociclib (CDKN2A) |
| 2192 | 14.0 | PTEN R130Q  FBXW7 R456H  PIK3CA E545D, R88Q  NRAS Q61R  CTNNB1 S33A | Yes  No  Yes  Yes  No | Yes  Yes  Yes  Yes  Yes | ABL2 P497fs*7  ATRX D1940fs*15  BLM N515fs*16  FBXW7 R465H  FGF6 V127M  JAK1 K860fs*16  JAK1 P430fs*2  MEN1 R521fs*7  MLL2 P2302fs*20  MLL3 K2797fs*26  MSH2 E48*  MSH2 Q324*  NOTCH1 R1586H  PIK3CA E545D  PREX2 S565fs*3  PTEN K267fs*9  PTEN R130Q  QKI A338T  SETD2 F636fs*6  SMARCA4 Q214  SMARCA4 T296fs*7  STK11 W332*  TET2 R1440fs*38  TET2 R550* | Unmatched by either ctDNA or tissue | Alternative clinical trial |
| 2348 | 4.0 | None | No | No | STK11 splice site 920+1G>A  TP53R273C | Unmatched by either ctDNA or tissue | Pembrolizumab |
| 2455 | 4.5 | KRAS G12D  PIK3CA N345K  KRAS amplification  FGFR2 amplification | Yes  Yes  Yes  Yes | Yes  Yes  Yes  Yes | KRAS G12D, amplification  PIK3CA N345K | Unmatched by either ctDNA or tissue | Pembrolizumab |
| 2456 | 4.4 | NF1 L298*  TP53 R273L  PIK3CA Amplification  FGFR2 Amplification  CCND2 Amplification  BRAF Amplification | Yes  No  Yes  Yes  No  Yes | Yes  Yes  Yes  Yes  Yes  Yes | Not done | Unmatched by either ctDNA or tissue | Pembrolizumab |
| 2555 | 33.6 | KRAS G12V | Yes | Yes | KRAS G12V | Matched by ctDNA | Trametinib (KRAS G12V), tamoxifen |
| 2618 | 25.8 | TP53 C238*  PIK3CA R88Q | No  Yes | Yes  Yes | Not done | Matched by ctDNA | Everolimus (PIK3CA), anastrozole |
| 2628 | 16.5 | TP53 V173M, R273H  PIK3CA Amplification  CCNE1 Amplification  MYC Amplification  MET Amplification  CDK6 Amplification  BRAF Amplification  CDK4 Amplification  ERBB2 Amplification  KRAS Amplification  EGFR Amplification  CCND2 Amplification  FGFR2 Amplification | Yes  Yes  No  No  Yes  No  Yes  Yes  Yes  Yes  Yes  No  Yes | Yes  Yes  Yes  Yes  Yes  Yes  Yes  Yes  Yes  Yes  Yes  Yes  Yes | FANCC N152fs*9  TP53 V173M | Matched by ctDNA | Bevacizumab (TP53)*, olaparib (FANCC), anastrozole |
| 2725 | 5.2 | TP53 H179R, R248W, R273H  BRCA1 E1346*  ERBB2 Y685* | No  Yes  Yes | Yes  Yes  Yes | Not done | Unmatched by either ctDNA or tissue | Adriamycin |
| 2760 | 32.9 | None | No | No | CCNE1 amplification  KRAS amplification  TP53 P250_I251del | Matched to tissue-based biopsy | Trametinib (KRAS), bevacizumab (TP53)*, carboplatin |
| 2774 | 11.1 | PIK3CA R88Q, P449T KRAS Q61H  ERBB2 R143Q | Yes  Yes  Yes | Yes  Yes  Yes | ARID1A G95fs*10  DNMT3A Q110fs*52  FUBP1 splice site 637-1G>A  KRAS Q61H  NFE2L2 D13G  PIK3CA P449T, R88Q,  PTCH1 S1203fs*52  SOX9 E50*, N96fs*156  TP53 Y126C | Matched by ctDNA | Everolimus (PIK3CA), anastrozole |
| 2889 | 2.1 | CDK6 Amplification  BRAF Amplification  MET Amplification  EGFR Amplification  CCNE1 Amplification | No  Yes  Yes  Yes  No | Yes  Yes  Yes  Yes  Yes | RB1 V654L  TP53 M160_A161insAIYK, Q165* | Matched by ctDNA | Sorafenib (BRAF), Trametinib (BRAF), Bevacizumab (TP53, EGFR) |
| 2919 | 31.3 | TP53 H168P  PIK3CA H1047Y  KRAS G12D | No  Yes  Yes | Yes  Yes  Yes | BRCA1 S1253fs*10  NOTCH1 R1586C  PIK3CA C378Y, H1047Y  SPOP M117V | Matched by ctDNA | Everolimus (PIK3CA), olaparib (BRA1) |
| 2974 | 3.9 | None | No |  | Not done | Unmatched by either ctDNA or tissue | Enzalutamide |
| 2992 | 7.3 | TP53 H179R, K132N  BRAF Amplification | No  Yes | Yes  Yes | BRCA1 K519fs*13  TP53 H179R | Matched to tissue-based biopsy | WEE1 inhibitor (BRCA1) |
| 2994 | 5.4 | None | No | No | ARID1A G276fs*87, T1514fs*13  CTCF G48fs*14, T204fs*18  HNF1A G292fs*25  INHBA R377H  KEL V340M  KRAS G13D  MLL2 G1235fs*95  PIK3CA N1044K, R88Q  PPP2R1A E216K  PTEN R130Q  QKI E135fs*5  RARA R294W | Matched to tissue-based biopsy | Everolimus (PIK3CA, PTEN) |
| 3000 | 5.3 | TP53 F270S | No | Yes | ESR1 amplification  NF1 loss exons 16-57  NF2 Y177fs*1  TP53 F270S | Unmatched by either ctDNA or tissue | Pembrolizumab |
| 3031 | 4.2 | TP53 C275G  CCNE1 Amplification | No  No | Yes  Yes | Not done | Unmatched by either ctDNA or tissue | Alternative clinical trial |
| 3044 | 5.7 | VHL S65A  TP53 V172F  CCND2 Amplification  PIK3CA Amplification | Yes  No  No  Yes | Yes  Yes  Yes  Yes | CDKN2A/B loss  NF1 truncation intron 14  TP53 splice site 993+1G>A | Unmatched by either ctDNA or tissue | Paclitaxel |
| 3062 | 5.8 | ERBB2 Amplification | Yes | Yes | Not done | Unmatched by either ctDNA or tissue | Navelbine |
| 3084 | 13.2 | TP53 R248Q, I195T | No | Yes | LRP1B A1555V  MSH6 D186fs*14  PIK3CA I102_E103>K  TERC amplification – equivocal  TP53 H179R | Matched by ctDNA | Everolimus (PIK3CA), bevacizumab (TP53)* |
| 3100 | 5.5 | AKT1 E17K  ARID1A Q1512* KRAS G12D  TP53 Y220C  MYC Amplification | Yes  No  Yes  No  No | Yes  Yes  Yes  Yes  Yes | AKT1 E17K  ARID1A Q1512*  CHD4 R1162W  KRAS G12D | Matched by ctDNA | Trametinib (KRAS), everolimus (AKT1) |
| 3173 | 6.6 | PTEN E242, D24V CTNNB1 S37F ARID1A S552*  KRAS V14I  TP53 V173L, Splice Site SNV  MYC Amplification | Yes  No  No  Yes  No  No | Yes  Yes  Yes  Yes Yes  Yes | ARID1A S552*  ASXL1 G646fs*12  CTNNB1 S37F  MYC amplification  PIK3R1 E403_I405del  PTEN E242* | Matched by ctDNA | Anastrozole, bevacizumab (TP53)*, celecoxib, everolimus (PTEN) |
| 3181 | 4.7 | APC R1920* | No | Yes | BRCA1 splice site 594-2A>C  CCNE1 amplification – equivocal  ERBB2 amplification  FBXW7 R658*  HGF amplification  PPP2R1A E216K, S256Y  TOP2A amplification – equivocal  TP53 L204fs*7, splice site 376-8_376delTCCTACAGT | Matched to tissue-based biopsy | Trastuzumab (ERBB2), pertuzumab (ERBB2) |
| 3222 | 17.0 | BRCA1 Exon 10 Deletion  TP53 Exon 7 Deletion | Yes  No | Yes  Yes | BRCA1 G1077fs*8  TP53 S241_N247del | Matched by ctDNA | Bevacizumab (TP53)*, olaparib (BRCA1) |
| 3289 | 3.7 | KRAS G12V | Yes | Yes | KRAS G12V  RNF43 R235fs*f182 | Matched by ctDNA | Trametinib (KRAS), pembrolizumab |
| 3332 | 9.9 | TP53 R342*, Q192fs, A276P | No | Yes | ATM R982fs*15  BCORL1 V676fs*51  BRCA2 G267*  CREBBP R75*  MAGI2 splice site 3031+1G>C  PIK3CA amplification – equivocal  PRKCI amplification  TERC amplification  TP53 R342* | Matched to tissue-based biopsy | Olaparib (BRCA2), nivolumab |
| 3336 | 23.0 | TP53 R342*, K132R  ARID1A Q1402*  EZH2 S644*  FGFR1 Amplification  BRAF Amplification | No  No  Yes  Yes  Yes | Yes  Yes  Yes  Yes  Yes | FBXW7 Y545C  LYN amplification – equivocal  MYC amplification  MYST3 amplification – equivocal  SDHA R31*  SPOP M117V  TOP1 amplification – equivocal  TP53 R342* | Matched by ctDNA | Regorafenib (BRAF), everolimus (FBXW7), cabozantinib |
| 3340 | 2.1 | TP53 N239, R248Q | No | Yes | FAT1 deletion exon 2, P2185fs*23  NF2 splice site 363+1G>T | Matched by ctDNA | Bevacizumab (TP53)*, embrolizumab (HPV+), denosumab |
| 3385 | 19.2 | None | No | No | CKDN2A/B loss | Matched to tissue-based biopsy | Palbociclib (CDKN2A), letrozole (ER+), doxorubicin, bevacizumab (VEGF positive on IHC) |
| 3417 | 2.3 | None | No | No | EGFR amplification | Unmatched by either ctDNA or tissue | Pembrolizumab |
| 3579 | 4.6 | TP53 R273H  CDKN2A R58* | No  Yes | Yes  Yes | CCND2 amplification – equivocal  CDKN2A p16INK4a R58*, p14ARF P72L  FGF23 amplification – equivocal  FGF6 amplification – equivocal  KDM5A amplification – equivocal  MYST3 R329W  TP53 R273H | Matched by ctDNA | Palbociclib (CDKN2A), bevacizumab (TP53)* |
| 3616 | 8.6 | TP53 Q165*, E285Q  PTEN Q214*, Q171*, amplification  MET amplification | No  Yes  Yes | Yes  Yes  Yes | Not done | Matched by ctDNA | Bevacizumab (TP53)*, pembrolizumab, ipilimumab |
| 3661 | 3.6 | TP53 Y220C | No | Yes | BRCA1 C61G  MYC amplification  PIK3CA amplification – equivocal  TP53 Y220C | Matched to tissue-based biopsy | Rucaparib (BRCA1) |
| 3713 | 23.2 | None | No | No | ARID1A Q2115fs*33  CHD4 R975H  CTCF S282fs*21  FGFR2 N549K  MLL3 S2123*  PIK3CA G1049R  PTEN K125N  TP53 Y163C | Matched to tissue-based biopsy | Lenvatinib (FGFR2), everolimus (PIK3CA, PTEN) |
| 3721 | 7.2 | TP53 Y234C, splice site SNV  PTEN T319fs, c.491_492+1del | No  Yes | Yes  Yes | Not done | Unmatched by either ctDNA or tissue | Alternative clinical trial |
| 3739 | 8.7 | TP53 G245V, R273C, c.559+2del | No | Yes | AKT2 amplification  AXL amplification  CCNE1 amplification – equivocal  ERBB2 amplification  FBXW7 R689W  FGF6 R43H  FGFR3 amplification – equivocal  PPP2R1A P179R  TP53 G245V | Matched by ctDNA | Bevacizumab (TP53)*, lapatinib (ERBB2), carboplatin |
| 3751 | 5.8 | None | No | No | BRCA1 Q1756fs*74  CTNNA1 E70fs*22  EP300 Q918fs*22  FAM123B R358*  LRP1B duplication exon 3-7  TP53 P152fs*14 | Matched to tissue-based biopsy | Rucaparib (BRCA1) |
| 3760 | 8.1 | TP53 C176Y, H193_I195del,  I195S, K139N  MYC Amplification  ERBB2 Amplification | No  No  Yes | Yes  Yes  Yes | EPHA3 amplification  ERBB2 amplification  MYC amplification – equivocal  TP53 C176Y | Matched by ctDNA | Pertuzumab (ERBB2), trastuzumab (ERBB2), lapatinib (ERBB2) |
| 3822 | 10.6 | TP53 V143M, R248Q | No | Yes | AURKA amplification  BRCA1 deletion exon 16  GNAS amplification  TP53 G245C  ZNF217 amplification | Matched to tissue-based biopsy | Niraparib (BRCA1) |
| 3824 | 3.9 | IDH2 R140Q | Yes | Yes | Not done | Unmatched by either ctDNA or tissue | Pembrolizumab |
| 3826 | 1.3 | TP53 R196*, R248Q  PIK3CA E542K  TERT Promoter SNV  CDKN2A R58*  CCND1 Amplification | No  Yes  No  Yes  No | Yes  Yes  No  Yes  Yes | Not done | Unmatched by either ctDNA or tissue | Alternative clinical trial |
| 3972 | 2.1 | TP53 Y220C, G245D  NOTCH1 R448*  AR V890M | No  No  No | Yes  Yes  yes | ERBB2 amplification  PIK3CA P104L  TP53 Y220C | Unmatched by either ctDNA or tissue | Adriamycin |
| 4002 | 15.5 | TP53 V272M, T230A  BRCA2 A1109fs  BRAF Amplification  PIK3CA Amplification  CDK6 Amplification  MET Amplification | No  Yes  Yes  Yes  No  Yes | Yes  Yes  Yes  Yes  Yes  Yes | NF1 duplication exons 2-4  RB1 splice site 1035_1049+2del17  TP53 V272M | Matched by ctDNA | Niraparib (BRCA2) |
| 4034 | 21.8 | NF1 L2395fs, L2390fs | Yes | Yes | ARFRP1 amplification – equivocal  PIK3CB D1067V  SPOP M117V  TP53 loss | Unmatched by either ctDNA or tissue | Alternative clinical trial |
| 4151 | 5.1 | PIK3CA H1047L  TP53 splice site SNV, Y220C, G244S  ERBB2 amplification  MYC amplification | Yes  No  Yes  No | Yes  Yes  Yes  Yes | FGFR4 D425N  MYC amplification  PIK3CA H1047L  TP53 splice site 993+1G>A  CCNE1 amplification | Unmatched by either ctDNA or tissue | Gemcitabine |
| 4164 | 14.1 | TP53 P278L  BRAF N581I  PIK3CA Amplification  ERBB2 Amplification  CCNE1 Amplification | No  Yes  Yes  Yes  No | Yes  Yes  Yes  Yes  Yes | AKT2 amplification  PIK3CA P104L  PPP2R1A S256Y  TP53 P278L | Unmatched by either ctDNA or tissue | Pembrolizumab |
| 4265 | 3.1 | TP53 G245S  NRAS G12D  PIK3CA K111E | No  Yes  Yes | Yes  Yes  Yes | BCL2L1 amplification  ERBB2 amplification – equivocal  MYST3 amplification – equivocal  PIK3CA K111E  PPP2R1A P179R  TERT promoter -124C>T  TOP2A amplification  TP53 G245S | Unmatched by either ctDNA or tissue | Alternative clinical trial |
| 4275 | 9.1 | NF1 R440*  TP53 T231fs | Yes  No | Yes  Yes | CCND2 amplification – equivocal  CCNE1 amplification  KDM5A amplification – equivocal  NF1 R440*  TP53 T231fs*9 | Matched by ctDNA | Trametinib (NF1), anakinra |
| 4316 | 20.0 | TP53 K132R, K120M  PIK3CA E545K, E542K  CDK6 Amplification  MET Amplification  PDGFRA Amplification  KIT Amplification | No  Yes  No  Yes  Yes  Yes | Yes  Yes  Yes  Yes  Yes  Yes | CDK6 amplification  FGFR4 amplification – equivocal  FLT4 amplification – equivocal  PDGFRB amplification – equivocal  TP53 K132R | Matched by ctDNA | Palbociclib (CDK6), lenvatinib (FGFR) |
| 4356 | 14.5 | KRAS G12A  ATM R337C | Yes  Yes | Yes  Yes | ACVR1B R485*  KRAS G12A | Matched to tissue-based biopsy | Nivolumab (TMB intermediate), ipilimumab (TMB intermediate) |
| 4396 | 17.3 | None | No | No | ATM G2695del  ERBB2 S310F | Matched to tissue-based biopsy | Trastuzuma, pertuzumab (ERBB2) |
| 4442 | 9.4 | EGFR G588S  KRAS G12A  ARID1A Y1389fs | No  Yes  No | Yes  Yes  Yes | ARID1A Y1389fs*56  ATM Q65*  FGF14 A236V  KRAS G12A  RB1 P777fs*33 | Matched by ctDNA | Trametinib (KRAS), olaparib (ATM) |
| 4475 | 10.4 | TP53 C135Y, R110P, C242F  CDKN2A W15* | No  Yes | Yes  Yes | ARFRP1 amplification – equivocal  BRCA2 splice site 7008-48_7008-2del47  CDKN2A p16INK4a W15*  HGF amplification – equivocal  KDM6A rearrangement intron 24  SMAD4 E161*  TP53 C242F | Matched to tissue-based biopsy | Olaparib (BRCA2) |
| 4488 | 17.9 | EGFR M793_L798del | Yes | Yes | Not done | Unmatched by either ctDNA or tissue | Bevacizumab, paclitaxel |
| 4504 | 1.7 | PIK3CA E545K, amplification  TP53 E286K  CDKN2A R58* | Yes  No  Yes | Yes  Yes  Yes | CDKN2A p16INK4a deletion, p14ARF deletion exons 2-, 3, p16INK4a R58*, p14ARF P72L  FAT1 S3005*  PIK3CA E545K  TERT promoter -124C>T  TP53 E286K | Matched by ctDNA | Palbociclib (CDKN2A), nivolumab (PDL1 IHC positive), everolimus (PIK3CA) |
| 4532 | 0.1 | TP53 P190T | No | Yes | Not done | Matched by ctDNA | Bevacizumab (TP53)*, pembrolizumab |
| 4573 | 3.9 | TP53 Q167fs | No | Yes | CDKN2A/B loss  MED12 G44V  POT1 CALD1-POT1 fusion  PTEN loss  TP53 Q167fs*14 | Unmatched by either ctDNA or tissue | Adriamycin, olaratumab |
| 4638 | 24.0 | None | No | No | BAP1 G185R | Matched to tissue-based biopsy | Olaparib (BAP1), letrozole, pazopanib |
| 4682 | 11.2 | TP53 G266R, E204*, I195N | No | Yes | Not done | Matched by ctDNA | Bevacizumab (TP53)*, atezolizumab |
| 4749 | 16.6 | TP53 R213*  PIK3CA amplification | No  Yes | Yes  Yes | Not done | Matched by ctDNA | Bevacizumab (TP53)*, carboplatin, paclitaxel |
| 4848 | 15.5 | TP53 Y220C | No | Yes | Not done | Matched by ctDNA | Bevacizumab (TP53)*, carboplatin, gemcitabine |
| 4909 | 8.1 | TP53 c773_782+19del | No | Yes | CDKN2A p16INK4a H83Y, p14ARF A97V  FBXW7 splice site 1122+1G>A  KRAS amplification  MUTYH Y165C  TP53 V122fs*26 | Matched to tissue-based biopsy | Trametinib (KRAS), palbociclib (CDKN2A) |
| 4914 | 14.4 | TP53 R175H | No | Yes | ATM splice site 6096-1G>C  CTNNB1 T297fs*22  LRP1B deletion exons 4-14  NFKBIA amplification  NKX2-1 amplification – equivocal  SDHA deletion exons 6-7  TP53 R175H  ZNF217 amplification – equivocal | Matched to tissue-based biopsy | Ipilimumab, nivolumab (TMB intermediate on tissue biopsy) |
| 4926 | 7.6 | TP53 R273C  PIK3CA K111E | No  Yes | Yes  yes | ARID1A P2005fs*10, splice site 2513-2A>G  ATM K2237fs*20  BRCA2 T3033fs*29  CCND1 V293G  CTCF T204fs*26  CTNNA1 R546*  ERBB3 N126K  EZH2 E225fs*1  FGFR3 V677I  FUBP1 S401fs*2  JAK1 P430fs*2  KRAS G12A  PIK3CA G106R, R93W  PTEN R130P  RNF43 G659fs*41  SMAD4 S32fs*1 | Matched to tissue-based biopsy | Olaparib (BRCA2, ATM), trametinib (KRAS), Nivolumab (MSI high, TMB high on tissue biopsy) |
| 4943 | 11.2 | KRAS G12D  FGFR2 Q609* | Yes  Yes | Yes  Yes | KRAS G12D  PIK3CA K111E | Matched to tissue-based biopsy | Temsirolimus (PIK3CA) |
| 4960 | 1.9 | PIK3CA E542K  TP53 R280T  MYC amplification | Yes  No  Yes | Yes  Yes  Yes | Not done | Unmatched by either ctDNA or tissue | Pembrolizumab |
| 4975 | 5.8 | FBXW7 R505G  PIK3CA E542K, E545K | No  Yes | Yes  Yes | BAP1 rearrangement exon 8  FBXW7 R505G | Unmatched by either ctDNA or tissue | Alternative clinical trial |
| 5081 | 10.2 | ARID1A A325fs  FBXW7 R465H  APC A2354fs | No  No  No | Yes  Yes  Yes | ARID1A A325fs*38  ARID2 Q1462*  BRCA2 Y600*  EP300 splice site 3671+1G>A  FBXW7 R465H  KMT2C (MLL3) W1671* | Matched to tissue-based biopsy | Olaparib (BRCA2), pembrolizumab |
| 5084 | 11.6 | TP53 R306* | No | Yes | AKT2 amplification  AXL amplification  CCNE1 amplification  HRAS amplification  IGF2 amplification  MYC amplification  PIK3CA E545K  PPP2R1A W257C  TP53 R306* | Matched by ctDNA | Bevacizumab (TP53)* |
| 5226 | 12.3 | None | No | No | AKT1 E17K  BCOR N1425S  CTNNB1 D32Y  EPHA3 E249K  FBXW7 R465H | Unmatched by either ctDNA or tissue | Megesterol acetate, tamoxifen, everolimus, clinoril, vandetenib |
| 5372 | 11.2 | TP53 G293fs  APC E1157fs | No  No | Yes  Yes | ACVR1B W316*  PPP2R1A S256F  TP53 G293fs*13 | Matched by ctDNA | Bevacizumab (TP53)*, everolimus (PPP2R1A), pembrolizumab (TMB intermediate on tissue biopsy), letrozole |
| 5436 | 10.8 | ATM R337C | Yes | Yes | Not done | Unmatched by either ctDNA or tissue | Pembrolizumab, avastin, letrozole |
| 5642 | 7.5 | TP53 N239D  CCNE1 amplification  RB1 R579* | No  No  No | Yes  Yes  No | PIK3CA H1065L  RB1 R579*  TP53 N239D | Matched by ctDNA | Bevacizumab (TP53)*, carboplatin, paclitaxel |
| 5695 | 6.9 | None | No | No | Not done | Unmatched by either ctDNA or tissue | Letrozole maintenance (No current disease) |
| 5696 | 8.9 | PIK3CA H1047R  TP53 R273H, V122fs | Yes  No | Yes  Yes | FGFR1 amplification  NSD3 (WHSC1L1) amplification  PIK3CA H1047R  TP53 V122fs*26 | Matched by ctDNA | Toptotecan, lenvatinib (TP53)* |
| 5697 | 1.8 | None | No | No | ARID1A R1528*  CTCF R49H  CTNNB1 S37C  FGFR2 S252W  PIK3R1 R649Q  PTEN R130G, V54fs*10 | Unmatched by either ctDNA or tissue | Arimidex |

† Overall survival was calculated from date of first ctDNA to date of last follow up or death.

†† If treatment included any agent targeted to ctDNA, it was considered a match by ctDNA. If treatment included only agents targeted to tissue-based sequencing, it was considered matched by tissue. If patient received only treatment that was unmatched by either ctDNA or tissue, it was considered unmatched treatment.

*Of note, TP53 alterations have been correlated with improved response to VEGF inhibition in multiple studies (16, 17, 19, 20), though this has not yet gained widespread acceptance.

**Abbreviations**: ctDNA, circulating tumor DNA; ID, identification number; OS, overall survival.
